# Full thickness 3D in vitro conjunctiva model enables goblet cell differentiation

**DOI:** 10.1038/s41598-023-38927-8

**Published:** 2023-07-28

**Authors:** Julian Schwebler, Christina Fey, Daniel Kampik, Christian Lotz

**Affiliations:** 1 Translational Center Regenerative Therapies (TLC-RT), Fraunhofer Institute for Silicate Research (ISC), Würzburg, Germany; 2 Chair of Tissue Engineering and Regenerative Medicine (TERM), University Hospital Würzburg, Würzburg, Germany; 3 Department of Ophthalmology, University Hospital Würzburg, Würzburg, Germany

**Keywords:** Biomaterials, Regenerative medicine, Tissue engineering, Differentiation, Biotechnology

## Abstract

In vitro culture and generation of highly specialized goblet cells is still a major challenge in conjunctival 3D in vitro equivalents. A model comprising all physiological factors, including mucus-secreting goblet cells has the potential to act as a new platform for studies on conjunctival diseases. We isolated primary conjunctival epithelial cells and fibroblasts from human biopsies. 3D models were generated from either epithelial layers or a combination of those with a connective tissue equivalent. Epithelial models were investigated for marker expression and barrier function. Full-thickness models were analyzed for goblet cell morphology and marker expression via immunofluorescence and quantitative real-time PCR. Simple epithelial models cultured at the air–liquid interface showed stratified multi-layer epithelia with pathologic keratinization and without goblet cell formation. The combination with a connective tissue equivalent to generate a full-thickness model led to the formation of a non-keratinized stratified multi-layer epithelium and induced goblet cell differentiation. In our model, a high resemblance to natural conjunctiva was achieved by the combination of conjunctival epithelial cells with fibroblasts embedded in a collagen-hydrogel as connective tissue equivalent. In the future, our conjunctival in vitro equivalent enables the investigation of goblet cell differentiation, conjunctival pathologies as well as drug testing.

## Introduction

The conjunctiva contributes to the tear film of the ocular surface by secreting smoothening mucus^1,2^. Crucial functions of the conjunctiva are protection from mechanical and pathogenic influences and the ability to react to harmful substances and allergens by IgE- and eosinophil-regulated mucus production^3^. Via this mechanism, the conjunctiva contributes to the privileged immune system of the ocular surface. Adverse influences like fluctuations in humidity, dry eye disease, allergens, infections, and auto-immune reactions, e.g., Stevens-Johnson syndrome or mucous membrane pemphigoid, can cause conjunctival destruction and eventually blindness^4,5^.

Interspersed in the non-keratinized epithelium of 2 to 9 layers of stratified squamous and columnar cells are highly specialized goblet cells. Their secreted mucins contribute to the tear film to ensure a smooth ocular surface. A high percentage of the inner part of goblet cells is filled with vesicles that contain mucus, while the cells’ nuclei are located on their basal side^6^. In general, mucins are tissue dependent and separated into two classes: membrane-associated and secreted mucins. In context of the conjunctival epithelium, the membrane-associated mucins MUC1, MUC4, and MUC16 as well as the secreted gel-forming mucin MUC5AC and the secreted soluble MUC7 have been identified^1,7^. Conjunctival goblet cells mainly express MUC5AC and to a lesser extent MUC16^1,8,9^. Conjunctival in vitro test systems using 2D and 3D culture addressing conjunctivitis, dry eye, pterygium fibroblast migration, fibrosis as well as drug delivery have been developed in recent years and have provided important information^10–13^. Complex 3D models that comprise the entire conjunctival structure have been developed by different authors, in which a connective tissue equivalent was used as scaffold for the culture of epithelial cells on top. In these studies, scaffolds made from Collagen I, Fibrin or decellularized conjunctiva were used, while Collagen I and Fibrin matrices included stromal cells^14–16^. These models were able to address specific questions of conjunctival research including differentiation, inflammation studies and clinical purposes. While mucus could be detected in these models via ELISA and Alcianblue/PAS stainings, explant cultures are difficult to standardize, and in vitro generated conjunctiva models display some but not all characteristics. Hence, there is still a need for a model that combines a multi-layered, stratified epithelium with goblet cells and specific MUC5AC staining inside their cup-like structures. Furthermore, the culture of these specialized goblet cells remains a major challenge due to missing or unknown key factors in their development^6^.

Therefore, animal tests are still being employed to investigate pathways of goblet cell differentiation and the pathophysiology of mucin-related diseases in general. Animal models of the conjunctiva comprise a broad variety of species such as mice, rats, guinea pigs, cats, rabbits, canines, and even non-human primates. The choice of animals depends on the physiological and biochemical properties as well as eye size^17,18^. Generally, biochemical characteristics, tear film and eye size similar to human tissue are preferred. Nevertheless, well-known differences persist between humans and other species, e.g., different Meibomian lipidomes and tear compositions^18–23^. There is a need to further develop in vitro solutions with key features such as goblet cell formation to model diseases and test drugs on highly predictive models. When fully developed, those in vitro models may tremendously improve drug development for various diseases, especially in the context of personalized treatments by using patient-derived cells. In the end, these in vitro tissues can be the steppingstone for tissue-engineered implants.

In conjunctival in vitro model development, goblet cell differentiation from epithelial cells and the detection of mucins are the most important and difficult challenges. Mechanisms behind this differentiation are not fully understood. Immune responses that increase IL-4, IL-13, and IgE levels are associated with an increase of MUC5AC expression in conjunctival epithelial cells, suggesting a role in the differentiation process to goblet cells^24–26^. Tian et al. showed that influencing Notch-signaling via gamma-secretase inhibitors may lead to goblet cell differentiation in a conjunctiva in vitro model^27^. Nomi et al. described that addition of growth factors, such as Keratinocyte Growth Factor (KGF) and Epidermal Growth Factor (EGF) is crucial for the differentiation processes of iPSC-derived conjunctival epithelial cells^28^. More generally, Tsai et al. showed that conjunctival fibroblasts are involved in goblet cell differentiation^15^. So far, the recreation of a complex conjunctiva model with sufficient goblet cell morphology and marker expression in a highly stratified multi-layer epithelium in vitro has not been achieved. The conjunctiva equivalent shown in this study displays ample goblet cell differentiation and marker expression, opening new approaches to decrypting factors involved in conjunctival goblet cell development. It also offers a novel in vitro model for the investigation of dry eye disease, which affects the number of conjunctival goblet cells^29^.

## Methods

### Human tissue

All experimental protocols were approved by the local ethics committee (Ethik-Kommission der Universität Würzburg, approval number 280/18sc). All experiments were conducted in compliance with the rules for investigation on human subjects, as defined in the Declaration of Helsinki. Human conjunctival biopsies were obtained with informed consent from patients of the University Hospital in Würzburg, Germany. These biopsies were from adult individuals receiving drainage surgery for glaucoma or scleral buckling surgery for retinal detachment, with no other ocular pathology. Specifically, the conjunctiva did not suffer from conditions like pterygium, neoplasia, pemphigoid, or significant inflammation. A total of 9 biopsies from different donors were used for this study.

### Isolation and culture of primary conjunctival cells

Primary conjunctival cells were isolated from single human tissue samples and were not pooled. Biopsies were incubated in a dispase solution (2 U/mL) for 1 h at 37 °C or overnight at 4 °C. After dispase digestion biopsies were transferred into PBS^-^ (without calcium chloride and magnesium chloride) for washing. Epithelial layers were gently scraped off with curved tweezers and transferred into a petri dish containing corneal epithelial cell basal medium (A1: Corneal Epithelial Cell Basal Medium + Corneal Epithelial Cell Growth Kit, American Type Culture Collection, ATCC + 1% Penicillin/Streptomycin (Sigma Aldrich, Darmstadt, Germany)). Remaining tissue was trypsinated for 2 min at room temperature (RT). Epithelial cells in A1 medium were centrifuged at 300×*g* for 5 min at RT and the supernatant removed. Remaining trypsinated tissue was as well centrifuged at 300×*g* for 5 min at RT and the supernatant was removed. Both samples were resuspended in 1 mL A1-medium. Prior to cell isolation, two separate T25 cell culture flasks with 2 mL of conjunctival fibroblast conditioned medium were incubated at 37 °C. Human conjunctival epithelial cells from scraping and trypsinating were then transferred into one T25 flask with a total volume of 3 mL.

For the isolation of primary human conjunctival fibroblasts, the remaining tissue was digested in a collagenase A solution (5 U/mL) at 37 °C for 1 h while vortexing the tube every 15 min after epithelial cell isolation. After digestion the tube was centrifuged at 300×*g* for 5 min at RT and resuspended in 2 mL DMEM (Thermo Fisher, Waltham, USA) + 10% FCS (Bio&SELL GmbH, Feucht, Germany) + 1% Penicillin/Streptomycin (Sigma Aldrich, Darmstadt, Germany), then seeded into a T25 culture flask.

For both, isolated epithelial cells and fibroblasts medium was changed every 2–3 days. Epithelial cells received 3.5 mL A1-medium and fibroblasts received 3.5 mL DMEM.

### Cell doubling time assay

Cells were seeded in a concentration of 4.500 cell/cm^2^ in a culture flask, cultured for four days, detached with Accutase (Thermo Fisher, Waltham, USA) and counted. Subsequently, cells were seeded in a new flask in the same concentration. Doubling times were calculated by the following formula^30^:

$$Doubling \, Time= \frac{duration*log (2)}{\mathrm{log}\left(Final \, Concentration\right)-\mathrm{log}(Initial \, Concentration)}$$

### Generation of conjunctival fibroblast conditioned medium

Conjunctival fibroblasts were cultured in a culture flask in DMEM. Medium was changed every 2–3 days. After reaching confluency, medium was changed every 24 h and the medium collected. This procedure was repeated for 4 days. Collected medium was centrifuged twice at 300×*g* for 5 min at RT and sterile filtered. Conditioned medium was stored at − 20 °C.

### Generation of reconstructed human conjunctival epithelial models (rhConE)

For the generation of rhConE, ~ 70% confluent epithelial cells were seeded into 24-well Brand-inserts (BRAND Insert 2in1, for 24 Well, Bio-CERT CELL CULTURE STERILE, Brand, Wertheim, Germany) in a concentration of 3 × 10^5^ cells (5 × 10^5^ cells/cm^2^) a volume of 300 µL PromoCell Keratinocyte Growth Medium 2 + SupplementMix + 1.5 mM CaCl_2_-solution (PromoCell, Heidelberg, Germany) (Pro2-medium). Cells were cultured in 2D up to passage 2 (p2), thus they were cultured in p3 in 3D models. After 2 h of adhesion time, 1.4 mL Pro2 medium was added to the well. An air–liquid interface (airlift) was set after 24 h. Airlift was seen as day 1 of model culture time in which rhConE received Pro2 medium + 73 µg/mL Ascorbic acid-2-phosphate (AA2P) + 10 ng/mL KGF (Pro3 medium) or Pro3 medium + 10% FibroLife medium (+ 2% FCS, without supplement LifeFactor, EGF-/TGF-β, Gentamicin and Amphotericin, Lifeline Cell Technology, San Diego, USA).

### Generation of full thickness conjunctiva models (FTConM)

For FTConM, 12 mm Snapwell™ Inserts with 0.4 μm pore polycarbonate membrane were used (Corning Incorporated, Corning, USA). As connective tissue equivalent, we used 700 µl of a compressed hydrogel of purified collagen I which was isolated from rat tails, and combined it with 4.5 × 10^4^ fibroblasts (6.43 × 10^4^ cells/mL), based on a study from Reuter et al.^31^. For a submerged culture, connective tissue equivalents received 250 µL DMEM apically and 5 mL DMEM basolaterally. After 4 days of submerged culture, all medium was siphoned, and 5.0 × 10^5^ epithelial cells were seeded on top of the connective tissue equivalent in 250 µL Pro2 medium. After 2 h of adhesion time, 2.5 mL Pro2 medium was added to the well. An airlift was set after 24 h and medium was changed to Pro10 medium. Medium was changed every 48–72 h.

### MTT-viability assay

On their respective days of culture (days 10, 15, and 20), a viability assay was performed on rhConE along with histological analyses. MTT (3-[4,5-dimethylthiazol-2-yl]-2,5 diphenyl tetrazolium bromide) is converted into formazan crystals by living cells, which is driven by mitochondrial activity^32^. The amount of living cells correlates with the color change from yellow to blue-purple during incubation with the reagent. Brand inserts were incubated basolaterally with 200 μL pre-warmed MTT solution. After 3 h of incubation at 37 °C, inserts were transferred into a new well. 500 μL isopropanol was added basolaterally and apically. The plate was set on a platform shaker for 1.5–2 h until all dye was extracted from the model. Subsequently, 1 mL of isopropanol was used to further rinse the models. In a 96-well plate, 200 µL were then analyzed by colorimetric measurement of extinction at 570 nm (Tecan plate reader M Infinite nano, Männedorf, Switzerland).

### Embedding and histological evaluation of rhConE and FTConM

For histological analyses on paraffin slides, rhConE and FTConM were fixated in Rotifix® (Carl Roth, Germany) for 2–3 h at RT. Subsequently, inserts were cut out, washed in dH_2_O and dehydrated via an ethanol/isopropanol series (50%, 70%, 80%, 96%, Isopronanol I, Isopropanol II). Remaining alcohol was removed via Xylene before probes were embedded in paraffin.

Using a microtome (HistoCore AutoCut, Leica, Wetzlar (Germany)), sections of 3.5 µm thickness were cut from paraffin-tissue blocks and transferred onto SuperFrost microscope slides (ThermoFisher Scientific, Waltham, USA). For further staining, sections were deparaffinized at 60 °C and immediately transferred into Xylene for rehydration followed by a descending ethanol series and dH_2_O (96%, 96%, 70%, 50%, dH_2_O). After staining sections with either Hematoxylin and Eosin (Morphisto, Offenbach am Main, Germany) or an Alcianblue-PAS, Kit (adaption: Mayer’s Hematoxylin instead of GILL III, Morphisto, Offenbach am Main, Germany), they were dehydrated by an ascending ethanol/isopronanol series and transferred into xylene (70%, 96%, Isopronanol I, Isopronanol II, Xylene I, Xylene II). Slides were then mounted with Entellan (Merck, Darmstadt, Germany) and glass coverslips.

For the evaluation of epithelial markers expressed by rhConE and FTConM, immunofluorescence was performed on paraffin sections and whole mounts. Paraffin sections were prepared as stated above, using Polysine™ adhesion microscope slides (epredia, Portsmouth, NH, USA). For whole mount stainings, FTConM were cut out of the inserts and fixated in Carnoy fixation until covered for 30 min at RT. Paraffin slides were blocked in 5% serum (donkey) for 20 min. Primary antibodies were applied and incubated at 4 °C overnight. On the following day slides were washed three times for 5 min with washing buffer. Secondary antibodies were applied and incubated at RT for 1 h. Slides were washed again three times and mounted with FluoroMount (Fluoromount-G with DAPI, Thermo Fisher, Waltham (USA)).

For whole mount stainings, models were cut out of their inserts and fixed in a 24-well plate. After fixation, models were treated with 0.2% Triton for 30 min. Subsequently, probes were washed for 5 min with washing buffer. 5% blocking solution was applied for 30 min at RT. Staining with primary and secondary antibodies was performed similar to paraffin slides. Instead of mounting, models were transferred into chamber slides and covered with FluoroMount. Primary antibodies: Cytokeratin 1 (Abcam, ab185628, dilution 1:600), Cytokeratin 13 (Abcam, ab92551, dilution 1:200), Cytokeratin 14 (Sigma-Aldrich, HPA023040, dilution 1:1000), Cytokeratin 19 (Abcam, ab7754, dilution 1:100), MUC5AC (Invitrogen, MA5-12178, dilution 1:100), Vimentin (Abcam, ab92547, dilution 1:2000), E-Cadherin (BD Biosciences, 610181, dilution 1:100). Secondary antibodies: AlexaFluor® 488, 555, 647 (dilution 1:400, LifeTechnologies, Carlsbad, CA, USA). Images were taken from representative areas for histological analyses.

### Impedance spectroscopy

Via impedance spectroscopy, transepithelial electrical resistance (TEER) was measured at a frequency of 1000 Hz to evaluate barrier quality and integrity of tissue models. For barrier evaluation, we defined TEER_1000 Hz_ values to give most precise information about the integrity of a multi-layer epithelium^33^. Tissue models were transferred into a 24-well Brand plate and received 600 μl of their respective impedance measurement medium basolaterally and apically. A 24-well TiN-measuring plate was used to analyze the amplitude and phase of the models by impedance analyzer LCR HiTESTER 3522-50 (HIOKI E.E. Corporation, Nagano (JP)). Results were displayed using LabVIEW software. After measurement, media was siphoned from the models which were then transferred back into their previous well-plates.

### Reverse transcription quantitative real-time PCR (RT-qPCR)

Cell-seeded scaffolds were lysed in TRK buffer supplemented with β-Mercaptoethanol, homogenized with a Tissue Lyser (Qiagen) and total RNA was extracted by using the peqGOLD MicroSpin Total RNA Kit (VWR) according to the manufacturer instructions. 1 µg of total RNA was reverse transcribed to cDNA using the i-Script Reverse transcription Supermix kit (Bio-Rad). Real-time quantitative PCR was performed with 1 µL cDNA using the SsoFAST™ EvaGreen® Supermix (Bio-Rad) and the CFX 96 Touch™ Real-time PCR cycler (Bio-Rad). All runs were performed at 60 °C annealing temperature and gene expression was set in relation to *GAPDH* (F-5’-GAAGGGCT CATGACCACAGT and R-5’-GGATGCAGGGATGATGTTCT) as reference gene. Primer sequences (Eurofins, Germany) used were *MUC5AC* (F-5ʹ-GTTTGACGGGAAGCAATACA and R-5ʹ-CGATGATGAAGAAGGTTGAGG) and *MUC16* (F-5ʹ-GCCTCTACCTTAACGGTT ACAATGAA and R-5ʹ-GGTACCCCATGGCTGTT GTG). Fold change of gene expression was assessed according to the ΔΔCT method.

### Statistical analysis

Statistical significance of MTT-assays, impedance spectroscopy and qRT-PCR was analyzed via two-way ANOVA tests. Values of *p* < *0.05* were considered to be significant. GraphPad Prism 8 software was used for statistical tests (GraphPad Software Inc., La Jolla, CA, USA).

## Results

### Isolated human primary conjunctiva cells retain growth and specific markers in vitro.

**Figure 1:**
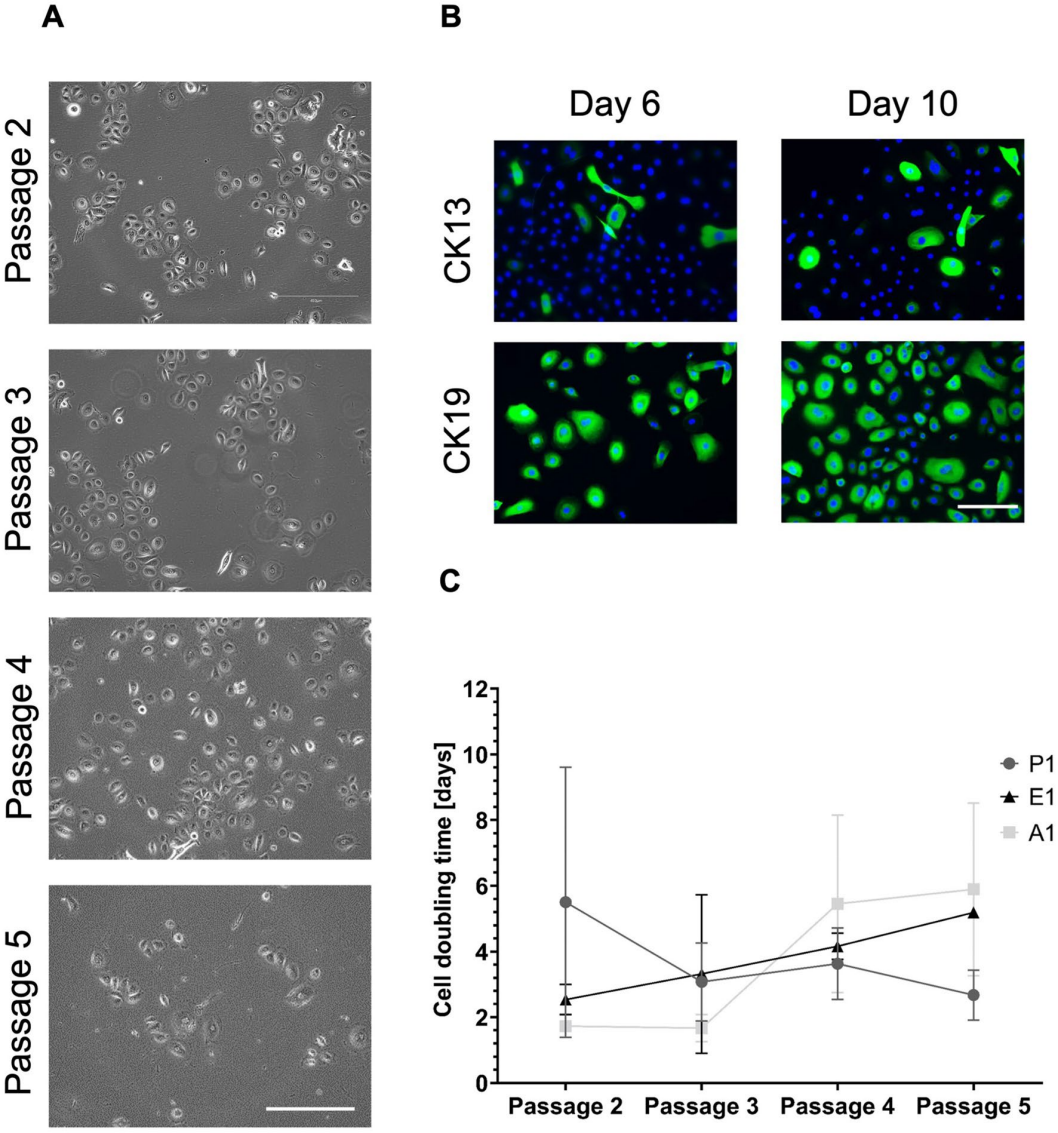
Human primary conjunctival epithelial cells retain specific markers and growth in vitro. Microscopic characterization of isolated human primary conjunctival epithelial cells and their doubling time in three different media. (**A**) Light-microscopic images of epithelial cells from passages (p) 2–5. Scale bar = 100 µm. (**B**) Immunofluorescence (IF) of cytokeratin (CK) 13 and CK19 performed on epithelial cells cultured on glass coverslips after 6 days and 10 days. Blue = DAPI, green = respective cytokeratin. Scale bar = 100 µm. (**C**) Cell doubling time graph of epithelial cells in three different media (Pro1, E1, A1) evaluated from p2 to p5 (mean and standard deviation, n = 3).

For the generation of conjunctival models, isolated epithelial cells were first characterized in a 2D environment, regarding their morphology and growth. Brightfield images of in Pro1-medium cultured epithelial cells were captured in passages (p) 1–5. Epithelial cells retained cobblestone-like morphology until p4 whereas cells showed more differentiated morphology in p5, characterized by losing their cobblestone-like morphology and demonstrating bigger and stretched cell shapes (Fig. 1A). Doubling time of epithelial cells was tested in three different media Pro1, E1 (EpiLife™ Medium (E) + human keratinocyte growth supplement (HKGS) + 1% Penicillin/Streptomycin) and A1. In earlier passages 2 and 3, epithelial cells showed the lowest doubling time when cultured in A1-medium, while cells in Pro1 and E1 showed a lower doubling time in passages 4 and 5 compared to A1 (Fig. 1C).

Conjunctiva specific cytokeratins (CK) 13 and 19 were positively stained via immunofluorescence after 6 days and 10 days of 2D culture while more cells were positive for CK19 than for CK13 (Fig. 1B).

### 3D cultured primary conjunctival epithelial cells develop a multi-layer epithelium

**Figure 2:**
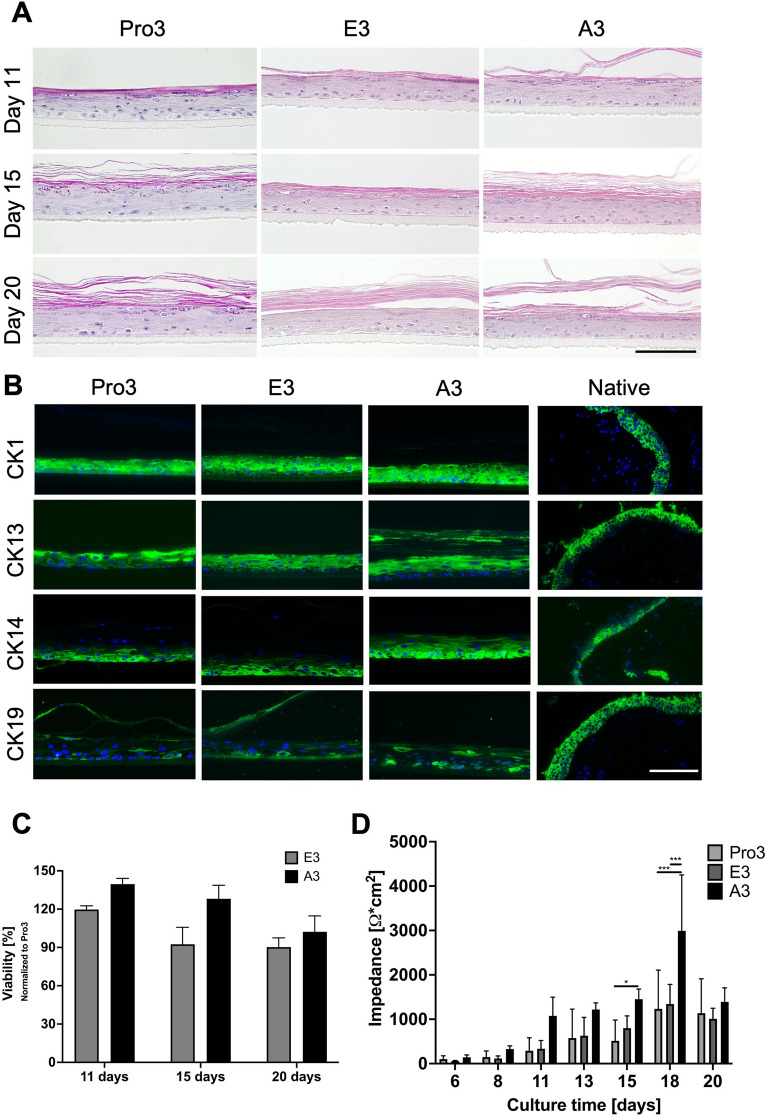
Conjunctival cells maturate at the air–liquid interface and form a multi-layered epithelium. Evaluation of different media on reconstructed human conjunctival epithelium (rhConE). (**A**) Histological analysis via hematoxylin & eosin (HE) stainings of rhConE cultured in Pro3, E3, or A3 medium after 11 days, 15 days, and 20 days. Scale bar = 100 µm. (**B**) IF of CK1, CK13, CK14, CK19 performed on rhConE cultured in Pro3, E3, or A3 after 15 days of culture. Blue = DAPI, green = respective cytokeratin. Scale bar = 100 µm. (**C**) MTT-assay of rhConE cultured in E3 or A3 medium normalized to models cultured in Pro3 (n = 3). (**D**) Measurement of transepithelial electrical resistance (TEER) 1000 Hz of rhConE cultured in Pro3, E3, or A3 from 6 days until 20 days of culture (n = 3). *p < 0.05, ***p < 0.001.

Conjunctival epithelial cells cultured under 3D conditions were investigated regarding their development of multi-layer epithelia and cell differentiation. Medium conditions used for the growth assay were also tested in 3D culture of epithelial cells with respective supplementation for 3D culture (+ CaCl_2_, + KGF, AA2P, see methods). After setting an air–liquid interface, rhConE were evaluated after 11 days, 15 days and 20 days via histology and MTT-assays. H&E stainings showed that rhConE cultured in all three medium formulations developed a multi-layer epithelium with stratified cells at every time point, together with keratinized layers (Fig. 2A). MTT-assays were normalized to Pro3 medium and showed no significant difference in viability between the different media at each time point of culture (Fig. 2C). Non-invasive impedance spectroscopy was performed on cultured models on multiple days throughout culture time, demonstrating the increasing barrier integrity of the models. Analyses showed a barrier increase in models of all tested conditions throughout culture time, while TEER_1000 Hz_ was highest when cultured in A3 medium. This effect was especially observable from 11 days (A3 = 1074 ± 424 Ω cm^2^, Pro3 = 291 ± 291 Ω cm^2^, E3 = 335 ± 186 Ω cm^2^), until it peaked at 18 days significantly (A3 = 2992 ± 1259 Ω cm^2^, Pro3 = 1233 ± 874 Ω cm^2^, E3 = 1344 ± 442 Ω cm^2^) (Fig. 2D). After 20 days of culture the model barriers of all conditions showed comparable TEER-values.

For further evaluation, conjunctiva specific CK13, CK19 as well as general epithelial markers CK1 and CK14 were investigated by IF after 15 days in culture (Fig. 2B). General epithelial markers CK1 and CK14 were positive for all tested medium formulations and time points. While conjunctiva specific CK13 was detected in all models, CK19 could only be detected in single cells for all tested media (Fig. 2B). Taken together, epithelial models developed a tight multi-layer epithelium together with specific marker expression. However, pathologic keratinization and no goblet cell differentiation could be observed in these models.

### The combination of connective tissue equivalent with epithelial cells induces goblet cell differentiation

**Figure 3:**
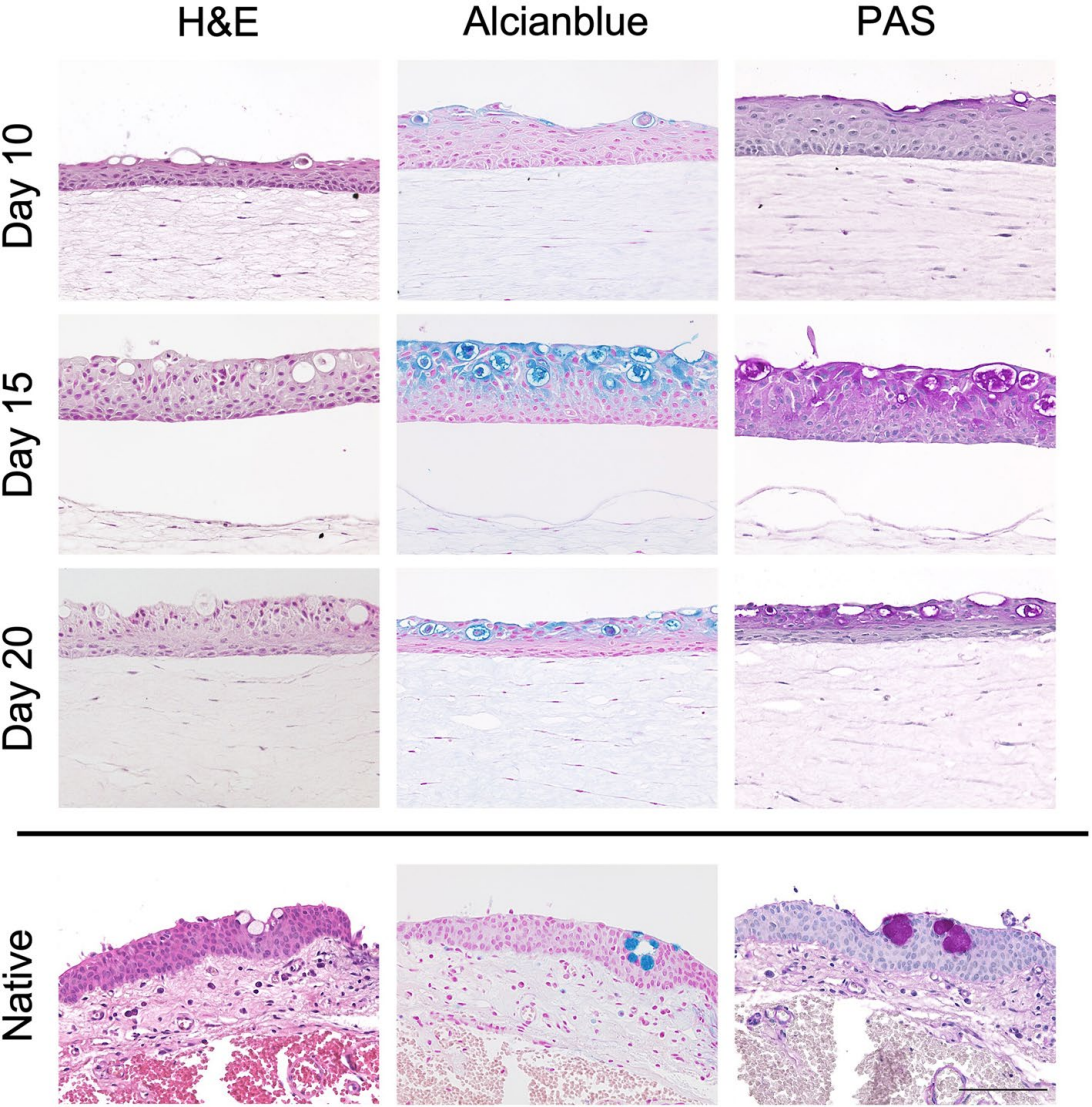
The combination of epithelial cells and connective tissue equivalent recreated in vivo anatomy. Histological analysis of full thickness conjunctival model (FTConM). H&E, Alcianblue and PAS stainings were performed on models after 10 days, 15 days, and 20 days and on native conjunctival tissue. Scale bar = 100 µm.

To recreate the conjunctival environment and reach physiological conditions, we generated a connective tissue equivalent by combining collagen type-I embedded fibroblasts with epithelial cells. This co-culture led to a stratified multi-layer epithelium of 7–9 cell layers, similar to the epithelial model alone, but without keratinization. In addition, conjunctival epithelial cells started widespread goblet cell differentiation, through which we generated the main cell types of the conjunctival epithelium. Goblet cells are characterized by their nucleus being located at one pole of a cell while the most volume is filled with mucus-granule^34^. This morphological characterization could be observed within FTConM while Alcianblue and periodic acid Schiff reaction (PAS) stainings revealed mucus in goblet cells being secreted on top of the epithelium. Both stainings comprise the optimal combination of PAS in which neutral muco-polysaccharides are stained purple while Alcianblue stains acidic mucus substances in light blue. An even distribution of fibroblasts was observed in the collagen matrix. From day 10 of culture, clear morphological differences indicating goblet cell differentiation were observed while Alcianblue and PAS revealed mucus secretion. After 15 days in culture, epithelial cells fully differentiated towards goblet cells, forming vesicles filled with mucus (Fig. 3). In summary, the introduction of a connective tissue equivalent induced goblet cell differentiation and led to 7–9 cell layers in the FTConM epithelium compared to the 4–6 in rhConE.

**Figure 4:**
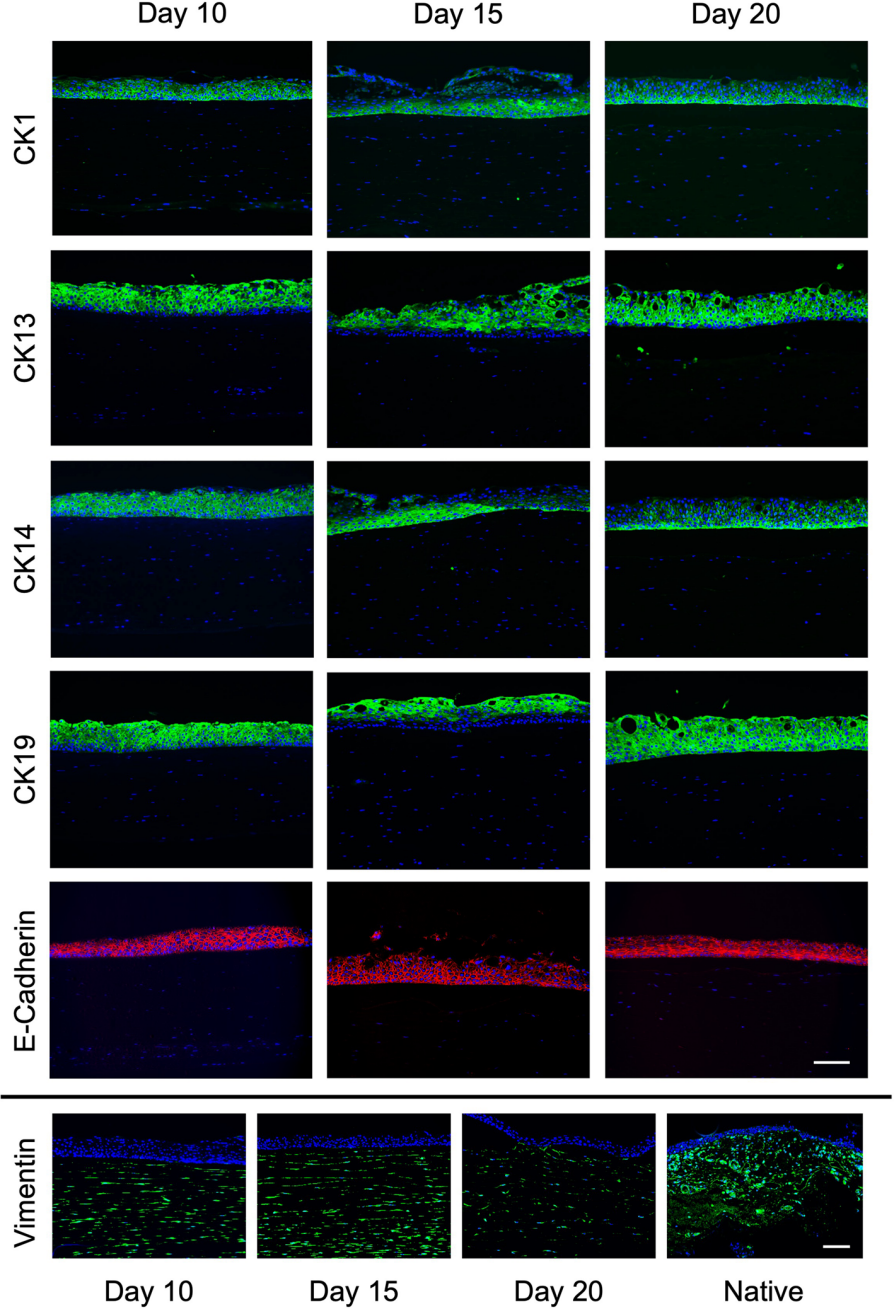
Full thickness conjunctival models (FTConM) displayed in vivo marker expression. Immunofluorescence staining of CK1, CK13, CK14, CK19, Vimentin, and E-Cadherin performed on FTConM after 10 days, 15 days, and 20 days of culture. Blue = DAPI, green = respective Cytokeratin/Vimentin, red = E-Cadherin. Scale bar = 100 µm.

Next, epithelial marker expression of FTConM was evaluated by immunofluorescence. Epithelial layers were stained for the keratin markers CK1, CK13, CK14 and CK19. All tested markers showed a positive signal throughout every tested time point of culture. With maturation of the model, CK1 and CK14 were mostly expressed in basal cells (Day 20) which resembles the expression of native tissue. Signals of conjunctiva specific markers CK13 and CK19 were detected throughout all epithelial layers and could be compared to those in native tissue (Fig. 2 vs Fig. 4). Immunofluorescence staining of CK13 and especially CK19 showed another important influence of the connective tissue equivalent. While rhConE showed CK19 expression only in single cells (Fig. 2B), epithelial cells in FTConM showed a high expression of CK19 at every tested time point (Fig. 4). Cell junctions were stained via E-Cadherin, indicating a cell barrier. In addition, fibroblast specific marker vimentin was stained positively for each maturation time and could as well be compared to native tissue (Fig. 4).

**Figure 5:**
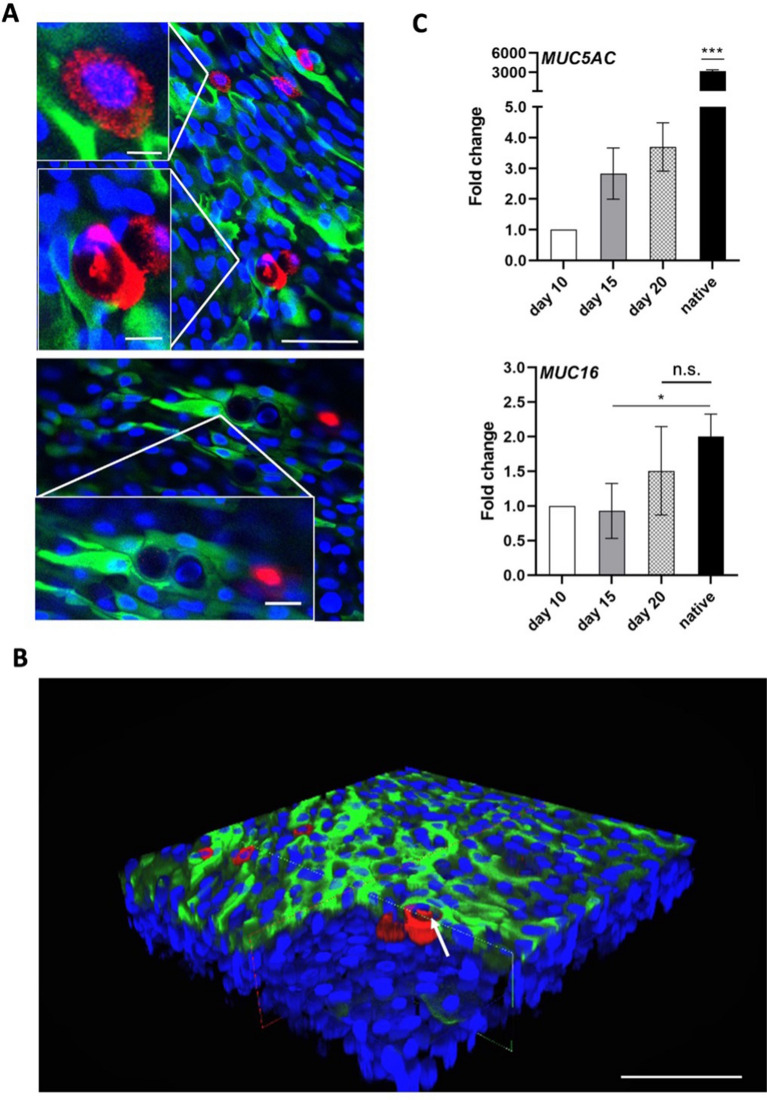
Full thickness conjunctival models (FTConM) induced goblet cell formation and mucus production. (**A**) Goblet cell formation in FTConM. Immunofluorescence staining of FTConM after 20 days of culture. Blue = DAPI, green = CK13, red = MUC5AC. Scale bar = 50 µm. Scale bar close-up = 25 µm. (**B**) 3D image of FTConM after 20 days. Blue = DAPI, green = CK13, red = MUC5AC. Arrow = goblet cell. Scale bar = 100 µm. (**C**) Quantitative real time PCR analysis of MUC5AC and MUC16 compared to native conjunctival tissue. Significant difference of MUC16 expression between 15 days and native tissue (n = 3, *p < 0.05). No significant difference between 20 days and native tissue (n = 3, p = 0.491).

While CK13 and CK19 are common markers for conjunctival epithelial cells, the most common marker for conjunctival goblet cells is MUC5AC, a gel-forming mucin that contributes to smoothening the ocular surface, which we investigated via whole mount immunofluorescence stainings and gene expression analyses. Confocal microscopy of whole mount stainings showed MUC5AC signal around nuclei and MUC5AC filled vesicles of goblet cells (Fig. 5A, Supplementary data S1). In addition, via the epithelium specific staining of CK13 and MUC5AC, it could be shown where the basal layer ends and the connective tissue starts (Fig. 5B, Supplementary data S1) since those markers are solely expressed in the conjunctival epithelium and not in the substantia propria.

Gene expression analysis of FTConM showed *MUC5AC* expression at all tested culture time points, while native tissue showed significantly higher expression than the model (Fig. 5C).

MUC16 is one of the main membrane-associated mucins of the ocular surface and was demonstrated by Gipson et al. to be expressed in conjunctival goblet cells^9^. Quantitative analysis showed a significant difference between the model´s expression at day 15 and native tissue (*p* < *0.05)*. However, a trend of increasing *MUC16* expression was observed after 20 days of culture in FTConM, showing no significant difference in expression levels compared to the native tissue (*p* = *0.491*). Taken together, we were able to demonstrate that the FTConM expressed the conjunctival goblet cell marker *MUC5AC* and *MUC16* on gene and protein level.

### Goblet cell differentiation is induced by the connective tissue equivalent

**Figure 6:**
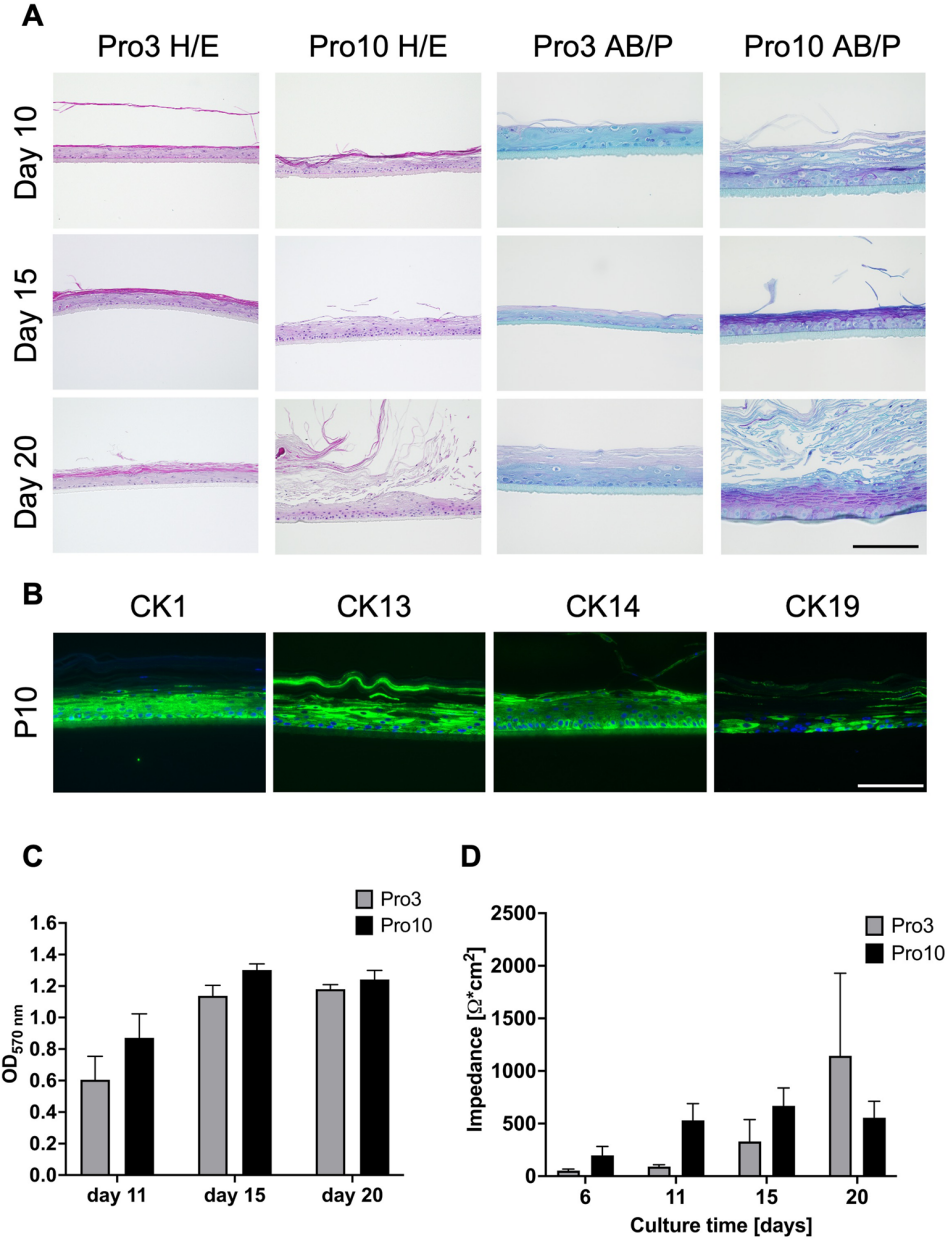
Medium supplementation on rhConE does not induce goblet cell differentiation. Media test of Pro3 vs. Pro10 on rhConE. (**A**) Histological analysis of in Pro3 or Pro10 cultured rhConE via H&E (left two columns) and AB/P (right two columns) after 10 days, 15 days, and 20 days of culture. (**B**) Immunofluorescence of CK1, CK13, CK14, and CK19 performed on rhConE cultured in Pro10 after 15 days. Blue = DAPI, green = respective cytokeratin. Scale bar = 100 µm. (**C**) MTT-assay of Pro3 vs. Pro10 cultured rhConE measured via optical density of 570 nm extinction (n = 3). (**D**) TEER_1000 Hz_ measurement of rhConE cultured in Pro3 or in Pro10 medium over the culture time from 6 days until 20 days (n = 3).

To demonstrate which factors are responsible for the induction of goblet cell differentiation in FTConM, three variables were analyzed when comparing to rhConE: Media, collagen, and presence of fibroblasts. To exclude the influence of medium, rhConE received Pro10-medium like FTConM. Compared to Pro3-culture, rhConE showed an increase in basal cells in Pro10-medium. Next to an increase of 1–3 cell layers, keratinization was observably increased in Pro10-cultured models. In addition, rhConE models cultured in Pro10-medium showed cells positive for Alcianblue and PAS in all epithelial layers, while models cultured in Pro3-medium demonstrated a less intense PAS staining (Fig. 6A). Immunofluorescence showed positive signals for CK1 and CK13 in all epithelial layers while CK14 was detected in basal cells. When cultured in Pro10-medium, rhConE showed increased CK19 expression in all layers compared to those cultured in Pro3-medium (Fig. 6B vs. Fig. 2B). MTT-assays confirmed viable cells in all tested models. In both tested conditions no significant impact on cell viability was observed (Fig. 6C). Throughout culturing time, measurement of TEER_1000 Hz_ showed barrier development independent of the employed medium (Fig. 6D). While TEER_1000 Hz_ values showed no significant differences throughout culture time, models cultured in Pro10 tended towards higher impedance at day 15 (Pro3 = 329 ± 208 Ω cm^2^ vs. Pro10 = 669 ± 170 Ω cm^2^) while models cultured in Pro3 showed higher impedance after 20 days (Pro3 = 1145 ± 208 Ω cm^2^ vs. Pro10 = 555 ± 156 Ω cm^2^). These results undermine the difference of Pro3 vs. Pro10 cultured rhConE in late culturing time points as seen in histological evaluation (Fig. 6A). Despite the higher PAS staining intensity, goblet cell differentiation was not achieved through culturing epithelial models in Pro10 medium, showing the significance of the connective tissue equivalent.

## Discussion

In this study, we developed a full thickness in vitro conjunctiva model that includes a multi-layer epithelium and mucus-secreting goblet cells. We used isolated primary epithelial cells from human conjunctival biopsies. Epithelial cells retained conjunctival epithelial markers CK13 and CK19 and were propagated up to passage 5 in 2D culture, enabling sufficient supply of cell material for model generation. The different tested media only displayed minor influence on the cells, demonstrating that HKGS (E-Medium), Bovine Pituitary Extract (Pro- and A-Medium) and Corneal Epithelial Growth Factor (A-Medium) are not tremendously changing the doubling time in 2D conjunctival epithelial cells. Neither did those factors contribute to goblet cell differentiation in 3D rhConE.

Epithelial cells developed a differentiated multi-layered epithelium, as indicated by an increasing barrier function during culture and protein expression of markers CK13 and CK19. H&E stainings revealed rhConE to build up to six cell layers, while top layers keratinized. In general, keratinization of conjunctival tissue in vivo is caused by pathologic events, e.g. by Vitamin A deficiency or Sjögren’s syndrome^35,36^. In our in vitro models, it is more likely that suboptimal culture conditions led to keratinization. Different media did not improve these non-physiological features, hinting at other missing cues prohibiting natural model tissue development. Therefore, supporting factors such as the extracellular matrix (ECM) and fibroblasts were included in the model, as it is known that differentiation and tissue development can be affected by those^37,38^.

To generate a reproducible connective-tissue equivalent, we used a compressed collagen-I hydrogel with embedded conjunctival fibroblasts in combination with epithelial cells. In FTConM, a multi-layer epithelium was observed, demonstrating mucus-filled and mucus-releasing goblet cells. At the same time keratinization was not observable anymore, indicating optimal culture conditions for the maturation of the tissue. Goblet cell differentiation was proven by morphology, Alcianblue and PAS stainings and MUC5AC detection, the most common marker of conjunctival goblet cells to be expressed and secreted^1^. Next to a qualitative evaluation of goblet cells, *MUC5AC* and *MUC16* were analyzed via RT-qPCR. Both markers were expressed in the model at all tested culture time points. For further comparison between rhConE and native tissue, cytokeratin expression of CK1, CK13, CK14, and CK19 was investigated on FTConM. While CK1 and CK14 were mostly expressed in basal cells with maturation of the model, CK13 and CK19 were expressed in all epithelial layers. In comparison to rhConE, detected signals of CK19 were clearly stronger in FTConM, which indicates FTConM to provide an environment that enables a more natural development of the tissue.

The comparison between rhConE and FTConM proves the importance of a connective tissue equivalent, which enabled goblet cell differentiation and thereby crucially improved our conjunctival model. Influencing factors that induced goblet cell differentiation can be narrowed down to three main components. Our connective tissue equivalent included collagen-I and conjunctival fibroblasts along with additional medium supplements. While rhConE received Pro3-medium, FTConM received Pro10-medium, which additionally includes fibroblast growth factor-2 (FGF2) and small amounts of fetal calf serum (FCS). In a study from 2020, Yokoo et al. showed that FGF2 can be involved in conjunctival goblet cell differentiation^39^. In rhConE, FGF2 supplementation in Pro10-medium did not induce goblet cell differentiation. On the other hand, with ongoing culture time, an increase in basal cells was observed in Pro10-cultured rhConE. Keratinized layers of Pro10 rhConE started to loosen and float off after 15–20 days of culture while cells were AB/P positive. These results suggest that media supplementation had an effect on rhConE but did not shift epithelial cells fully towards goblet cell differentiation.

In a recent study, Nomi et al. Showed that Keratinocyte Growth Factor (KGF) is crucial for the development from human iPSC to conjunctival epithelial cells, especially goblet cells^28^. In our rhConE model, a KGF supplementation alone did not induce this effect, indicating that further factors are needed for primary cells. Co-cultured fibroblasts could also have a major impact on differentiation. Tsai et al. showed that the use of conjunctival fibroblasts in a collagen-I gel led to Alcianblue and PAS-positive cells including goblet cell-like morphology while a co-culture with 3T3-cells led to a higher layer epithelium but not to Alcianblue and PAS-positive cells^15^. These results suggest conjunctival fibroblasts to express factors that induce goblet cell differentiation. The positive effect of co-cultured fibroblasts in a collagen-hydrogel could also be demonstrated in our FTConM. These findings suggest that a combination of supplementary and fibroblast secreted growth factors and ECM is important to recapitulate the natural development of conjunctival tissue. In our model we achieved an environment that allows such natural development. Therefore, the key factors of goblet cell differentiation can be further decrypted. In addition, our model may provide a tool for new opportunities for the reduction of animal testing of conjunctival diseases.

The combination of a multi-layered epithelium that includes goblet cells with cup like structures and MUC5AC expression emphasizes the physiological homeostasis and differentiates it from the other in vitro conjunctival models. Current conjunctival in vitro models show specific markers expression such as CK19 and MUC5AC. However, a lack of cup-like structures filled with mucus and specific MUC5AC staining or the display of a multi-layer epithelium after differentiation implicate missing cues for its natural physiology^14,27,40^. The combination of all physiology resembling factors in an in vitro model is challenging. Witt et al. were able to achieve those in an in vitro human conjunctival explant model on decelullarized porcine conjunctiva^16^. Using the explant, AB/P positive goblet cells were observable, even though MUC5AC could only be stained on single spots in the model. These results were not reproducible, using isolated epithelial cells on the scaffold instead of an explant^16^. Nonetheless, the explant model is promising for its clinical purposes addressing regenerative methods for conjunctival scarring. Yet, for the investigation of conjunctival diseases in vitro, an explant free model would be more suitable due to the better reproducibility and obtainment of the connective tissue equivalent, based on a defined collagen hydrogel. Once all key factors have been decrypted that shift conjunctival epithelial cells in 3D culture into goblet cell differentiation, those culture conditions can be adopted in various research fields of conjunctival in vitro culture and bring 3D models one crucial step closer to physiological resemblance and thus, to higher predictivity of drug testing and disease modelling. Further, models for the investigation of conjunctival fibrosis after glaucoma surgery are being developed and investigated. Most recently, a tenon`s capsule/bulbar conjunctiva interface model containing macrophages has been developed by Kozdon et al. and was tested on its mechanical properties^41^. In combination with our model, such experiments could be further developed with a full functional epithelium to analyze the interaction of fibrosis with epithelial cells in a 3D in vitro model.

In our study we were able to combine all conjunctiva specific factors, including stratified multi-layer epithelia containing goblet cells. The expression of markers specific to conjunctival epithelial cell differentiation was confirmed at the level of protein (immunofluroescence) and mRNA (RT-qPCR). We were able to show MUC5AC filled cups of goblet cells via confocal microscopy. Due to the use of a stromal equivalent our model increases reproducibility and applicability of conjunctival in vitro studies. In the future, this model could be employed for the investigation of different causes of conjunctival diseases in order to provide a highly predictive, human derived base for clinical studies with the additional benefit of reducing animal experiments.

## Supplementary Information

Supplementary Video 1.

Supplementary Figures.

## Data Availability

The datasets generated during and/or analyzed during the current study are available from the corresponding author on reasonable request.
