# Supplementary material for: Full thickness 3D in vitro conjunctiva model enables goblet cell differentiation
Source: Sci Rep. 2023 Jul 28;13:12261. doi: 10.1038/s41598-023-38927-8 (PMC10382544; doi:10.1038/s41598-023-38927-8)
Supplement: Supplementary file 2 — Supplementary Figures. [file 41598_2023_38927_MOESM2_ESM.docx]

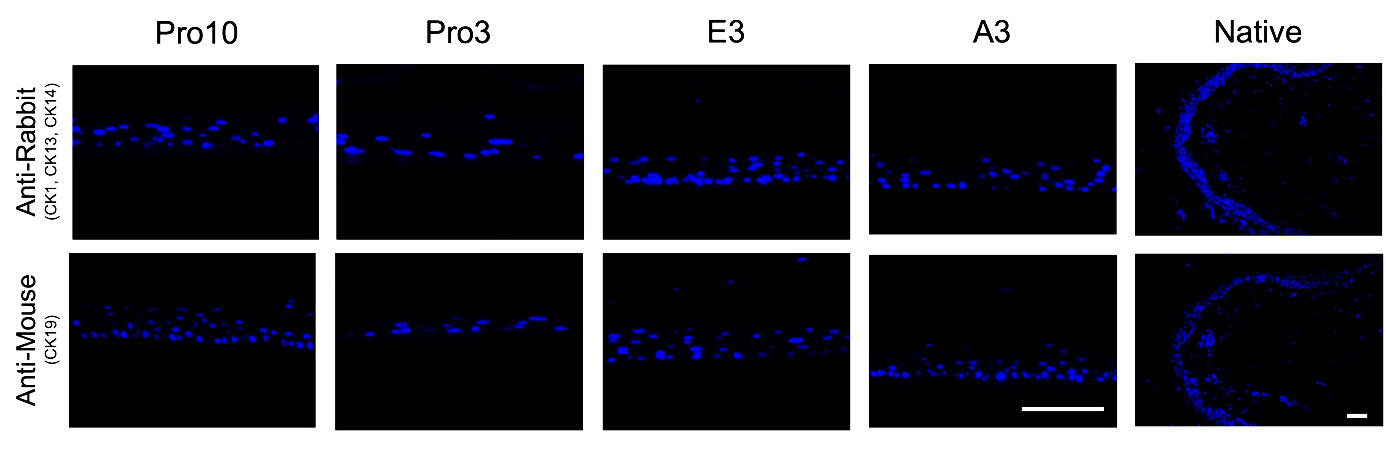


Supplementary Figure 1 - Immunofluorescence negative controls (DAPI + secondary antibody only) of reconstructed conjunctival epithelial models and native tissue for Anti-Rabbit (CK1, CK13, CK14) and Anti-Mouse (CK19) secondary antibodies. Channels of DAPI and the respective secondary antibody are merged. Scale bars = 100 µm.


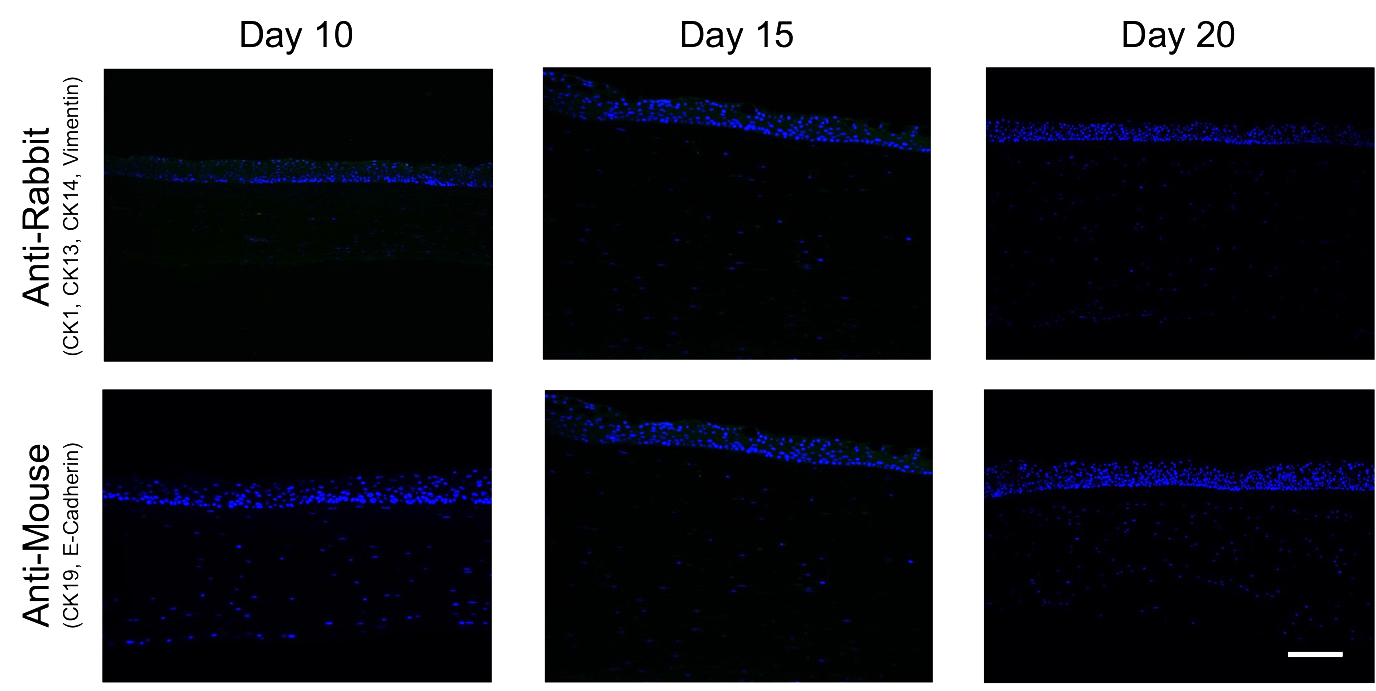


Supplementary Figure 2 - Immunofluorescence negative controls (DAPI + secondary antibody only) of full thickness conjunctiva models for Anti-Rabbit (CK1, CK13, CK14, Vimentin) and Anti-Mouse (CK19, E-Cadherin) secondary antibodies. Channels of DAPI and the respective secondary antibody are merged. Scale bars = 100 µm.


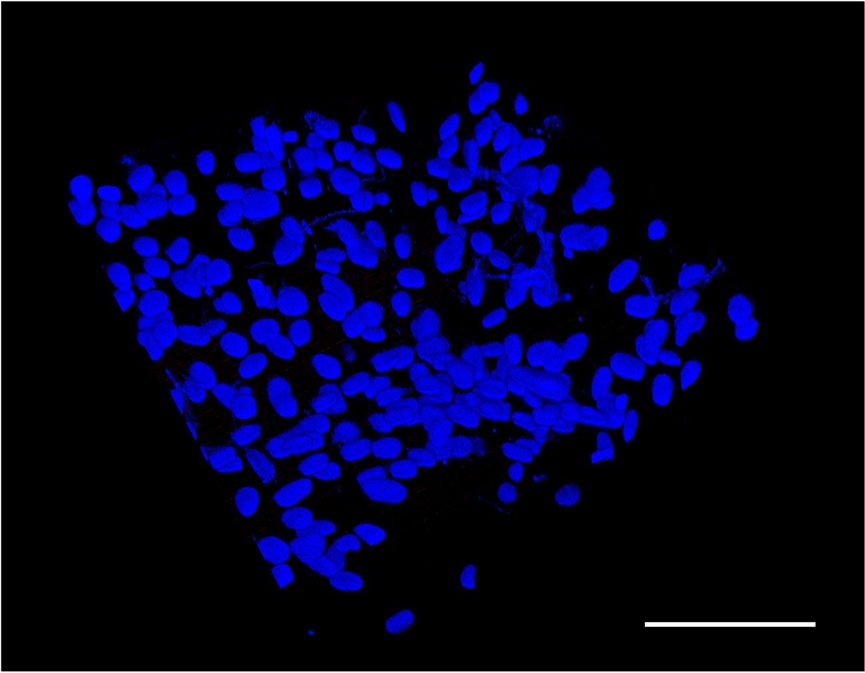


Supplementary Figure 3 - Immunofluorescence negative control (DAPI + secondary antibodies only) of FTConM whole mount staining for Anti-Rabbit (CK13) and Anti-Mouse (MUC5AC). Channels of DAPI and respective secondary antibodies are merged. Scale bar = 100 µm.
